# Faradisation in Asphyxia from Chloroform

**Published:** 1860-07

**Authors:** 


### ARTICLE XXIV.

### *Faradisation in Asphyxia from Chloroform.* By Dr. Friedberg.

A boy, aged four years, inhaled chloroform from a sponge prior to undergoing an operation for the removal of a small tumor of the lower eyelid. At most ʒj. was employed, and in less than two minutes alarming appearances were produced. The pulse had become very small, the respiration consisted only in a short, rattling inspiration, the face was livid, and the limbs were relaxed. Windows were opened, cold water was sprinkled on the face, ammonia was applied to the nostrils, and a small sponge was carried down to the epiglottis, in order to remove any mucus and to endeavor to excite coughing—the thorax being at the same time rubbed, and sometimes dashed with cold water. These might have been employed for two or three minutes, when a further change in the child's condition was observable The pulse had now quite ceased, the countenance was that of a corpse, and the lower jaw had dropped. When the eyelids were separated to examine the pupils (which were dilated) they remained gaping. As no time was evidently to be lost, the author had recourse to artificial respiration. He did not endeavor to induce this, however, by the insufflation of air, regarding that as a very uncertain procedure. The methodical compression of the abdomen is a much better one, and was executed. While an assistant compressed the abdomen with both his hands beneath the navel, in order to prevent the viscera receding below, the author pressed the upper portion of the abdominal walls towards the diaphragm, removing the hands then immediately, in order to allow of the expansion of the lungs. This rhythmical procedure was kept up for about three minutes without any appreciable advantage. A complete relaxation of the diaphragm, in fact, existed, as there was neither resistance offered by it to the passage of the hand or any subsequent vaulting of the epigastrium. It was now resolved to Faradise the diaphragm, in order to induce its contraction. One of the conductors of Bois Reymond's induction apparatus was applied over the phrenic nerve (where the omo-hyoideus lies at the outer edge of the sterno-cleido-mastoideus,) and the other to the seventh intercostal space, pressing this latter deeply towards the diaphragm. The Faradisation was performed sometimes on one side and sometimes on the other, the stream being interrupted ten times on the contraction of the diaphragm, giving rise to vaulting of the epigastrium, a short sob occurring at the same time. The Faradisation being now suspended, a slight spontaneous inspiration occurred, which was followed by a second and third, and a temporary reddening of the face, the pulse also becoming perceptible. Compression of the abdomen was again resorted to, the tension of the diaphragm now offering its proper resistance. The attempt to suspend the compression at the end of ten minutes of its employment was attended with an immediate enfeeblement of the respiration and pulse. It was therefore resumed for another ten minutes, the extremities being also rubbed, the face sprinkled with water, and ammonia applied to the nose. The recovery at last became so complete, that the operation was proceeded with and the child did very well.—*Virchow's Archiv.*

